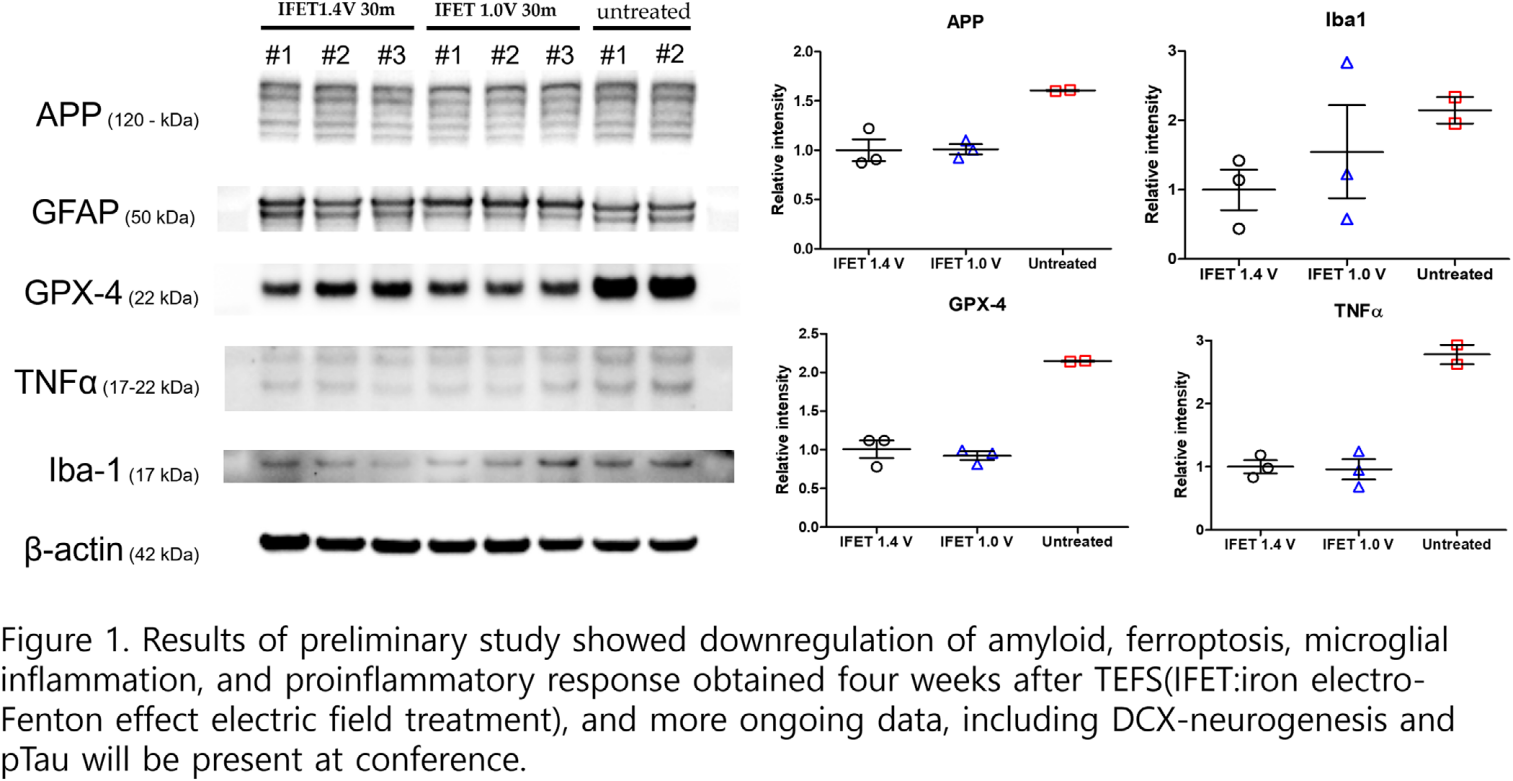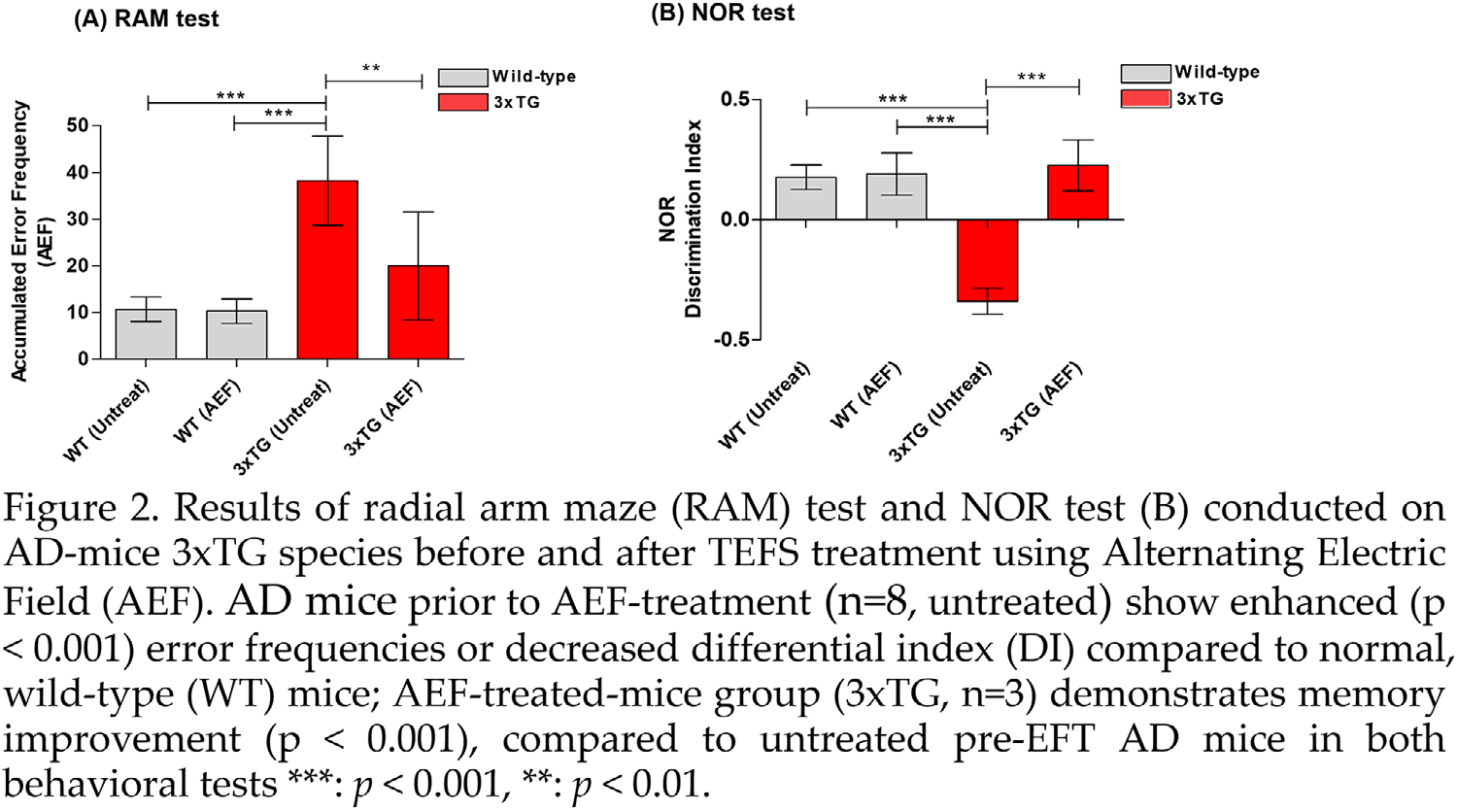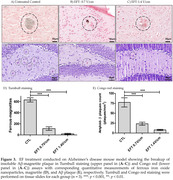# Transcranial electric field stimulation on iron deposition cures multiple pathologies of Aß, tau, microglial inflammation and improves cognitive impairment in Alzheimer’s mouse model

**DOI:** 10.1002/alz.095191

**Published:** 2025-01-09

**Authors:** Jong‐Ki Kim

**Affiliations:** ^1^ Daegu Catholic University, Daegu, Daegu Metropolitan City Korea, Republic of (South)

## Abstract

**Background:**

The interplaying neuropathology of amyloid plaque, tau tangles, and microglia‐driven inflammation (tri‐pathology) are related to neuronal and synaptic loss damage in Alzheimer’s damages. Interventions that target Aβ or tau individually have not yielded substantial breakthroughs. Iron plays a pivotal role in tri‐pathology by protein‐bound iron‐oxide deposition in amyloid plaque, tau tangle, and microglia, resulting in redox‐active toxicity or microglial response induction, such as proinflammatory activation, autophagy dysfunction, and ferroptosis.

**Methods:**

Using a single transcranial treatment, we stimulated the iron deposition in the tri‐pathologic iron inclusions. This treatment involved the use of a noncontact capacitive‐electrode alternating electric field (1 Mhz, < 1.5 V/cm) (TEFS) to induce the electro‐Fenton effect from the iron oxide surface of pathologic iron‐inclusion bodies in the brain of an Alzheimer’s disease (AD) mouse model (3xTg). We assessed the therapeutic effect of this phenomenon one month after treatment using histology, western blot analysis, and behavioral tests and compared the results with an untreated control.

**Results:**

Our study revealed that TEFS‐mediated depletion of iron deposition and Aβ plaque prevented tau expression in early‐stage AD. In late‐stage AD, TEFS depleted Aβ plaque and tau tangle and downregulated ferroptosis and microglial‐driven neuroinflammation. Furthermore, TEFS improved impaired cognitive function, restored hippocampal neurogenesis, and altered microglial phenotype in both stages of AD brain.

**Conclusion:**

Our results suggest that TEFS‐driven degradation of insoluble iron deposition cures multiple tri‐pathologies, promotes disease‐modifying properties, and offers a potential molecular‐targeting nonpharmacological treatment option for AD.